# Health-Related Quality of Life Evaluation Using the Short Form-36 in Patients With Human T-Lymphotropic Virus Type 1-Associated Myelopathy

**DOI:** 10.3389/fmed.2022.879379

**Published:** 2022-04-11

**Authors:** Miyuna Kimura, Junji Yamauchi, Tomoo Sato, Naoko Yagishita, Natsumi Araya, Satoko Aratani, Kenichiro Tanabe, Erika Horibe, Toshiki Watanabe, Ariella Coler-Reilly, Misako Nagasaka, Yukari Akasu, Kei Kaburagi, Takayuki Kikuchi, Soichiro Shibata, Hirofumi Matsumoto, Akihito Koseki, Soichiro Inoue, Ayako Takata, Yoshihisa Yamano

**Affiliations:** ^1^Department of Anesthesiology, St. Marianna University School of Medicine, Kawasaki, Japan; ^2^Department of Rare Diseases Research, Institute of Medical Science, St. Marianna University School of Medicine, Kawasaki, Japan; ^3^Division of Neurology, Department of Internal Medicine, St. Marianna University School of Medicine, Kawasaki, Japan; ^4^LSI Medience Co., Tokyo, Japan; ^5^Department of Frontier Medicine, Institute of Medical Science, St. Marianna University School of Medicine, Kawasaki, Japan; ^6^Department of Practical Management of Medical Information, St. Marianna University Graduate School of Medicine, Kawasaki, Japan; ^7^Division of Hematology and Oncology, Department of Medicine, University of California, Irvine School of Medicine, Orange, CA, United States; ^8^Department of Neurology, Yaizu City Hospital, Yaizu, Japan; ^9^Department of Preventive Medicine, St. Marianna University School of Medicine, Kawasaki, Japan

**Keywords:** HTLV-1, SF-36, SF-6D, quality of life, HTLV-1-associated myelopathy

## Abstract

**Background:**

Human T-lymphotropic virus type 1 (HTLV-1)-associated myelopathy (HAM) is a neuroinflammatory disease, causing various neurological symptoms, including motor, sensory, and bladder and bowel dysfunctions. This study was designed to reveal the impact of HAM and related symptoms on health-related quality of life (HRQoL).

**Methods:**

We analyzed the Short Form-36 (SF-36) and clinical data of 538 patients with HAM registered in the HAM-net, a nationwide patient registry for HAM in Japan. HRQoL was evaluated using the SF-6D (a health state utility value calculated from the SF-36) and eight SF-36 subscales. A general liner model was used to estimate the impact of major HAM-related symptoms, including gait dysfunction, sensory disturbance in the legs (pain and numbness), urinary dysfunction, and constipation, on the SF-6D and SF-36 subscale scores.

**Results:**

The mean age and disease duration were 62.0 and 16.5 years, respectively. Of the patients, 73.2% needed walking aid; 42.7 and 67.1% had leg pain and numbness, respectively; 92.1% had urinary dysfunction; and 77.9% had constipation. The mean SF-6D score was 0.565, which was significantly lower than the national average (0.674 in the 60–69 years age group; *p* < 0.001), exceeding the minimal important difference (0.05–0.1). All the major symptoms were significantly associated with a decrease in the SF-6D score. The SF-36 subscale scores were significantly lower than the national standard of 50 (*p* ≤ 0.001), except for mental health (MH). Gait dysfunction was associated with lower scores in physical functioning (PF), limitations on role functioning because of physical health, bodily pain, general health perception (GH), vitality (VT), and social functioning; however, no association was observed between gait dysfunction and limitations on role functioning because of emotional problems and MH. Meanwhile, sensory disturbance in the legs was associated with a decrease in scores in all subscales. Urinary dysfunction was associated with worse PF, GH, VT, and MH. Constipation was associated only with PF.

**Conclusion:**

HRQoL of patients with HAM was worse than that of the general population and was associated with all major symptoms. Thus, patients should be comprehensively managed to achieve better HRQoL.

## Introduction

Human T-lymphotropic virus type 1 (HTLV-1) is estimated to infect at least 5–10 million individuals with a heterogeneous global distribution (1). Its endemic regions include the Southwestern part of Japan, South America, the Caribbean area, and sub-Saharan Africa. This retrovirus causes a neuroinflammatory disease, called HTLV-1-associated myelopathy (HAM), in a small proportion of infected individuals (2, 3). Due to the chronic inflammatory destruction of the spinal cord, HAM results in various symptoms, including motor and sensory disturbance in the legs and neurogenic bladder and bowel dysfunctions (4). In severe cases, patients become wheelchair-bound and require urinary catheters and enemas. Although corticosteroids are most widely administered to slow the disease progression, no curative therapy is currently available (5). Thus, symptomatic treatment is the mainstay of management. Therefore, data regarding the burden of complicated symptoms on health-related quality of life (HRQoL) are necessary to enhance medical care and research for patients with HAM. However, information is extremely limited because of the rarity of the disease.

Among the HRQoL measures, the Short Form-36 (SF-36) and the EuroQoL 5-dimension (EQ-5D) are most widely used to measure generic health status in clinical research (6, 7). The SF-36 assesses physical and mental health status using 36 questions. The Short Form 6-dimension (SF-6D), which is a health state utility index, can be calculated from the SF-36 in some languages (8, 9). The EQ-5D also generates a health state utility index using five questions (10). Regarding HRQoL of patients with HAM, several reports from Brazil have been published. Mainly using the SF-36, they have shown that this patient population had poor HRQoL (11–17). Although the SF-6D has not been reported to date, Rosadas et al. have recently demonstrated that the EQ-5D index in patients with HAM living in Brazil and the United Kingdom was significantly lower than that in the general population and HTLV-1 carriers (18). Moreover, these studies have reported that HAM-related symptoms, such as gait dysfunction, pain, and lower urinary tract symptoms, decreased HRQoL by comparing patients with each symptom with those without each symptom (11–18). However, as HAM simultaneously causes multiple symptoms, strictly estimating the effect of each symptom is impossible using univariate analysis performed in these studies with relatively small sample sizes (<60 subjects).

Therefore, this study analyzed the SF-36 and SF-6D data obtained from more than 500 patients in Japan to reveal the effects of HAM and related symptoms on HRQoL. These patients had worse HRQoL than the general population. Moreover, multivariate analysis demonstrated that not only gait dysfunction but also other symptoms of HAM were significantly associated with poorer HRQoL. To our knowledge, this is the first study that has examined the SF-36 and SF-6D of patients with HAM from Japan.

## Materials and Methods

### Data Source and Study Population

**FIGURE 1:**
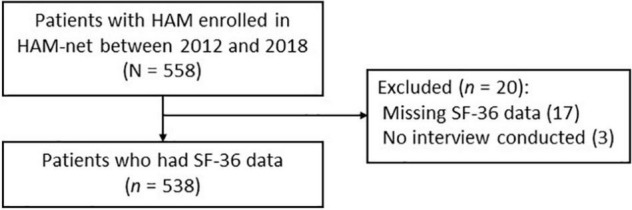
Study flow chart. HAM, HTLV-1-associated myelopathy.

We used data from the “HAM-net,” a nationwide patient registration system for HAM in Japan (UMIN000028400) (19). The HAM-net recruits patients from all over Japan through the website or leaflets distributed to patients at clinics and patient meetings. Patients can apply to the HAM-net office via telephone, fax, or e-mail. After registration, trained nurses and coordinators uniformly conduct annual telephone interviews with patients to collect their data, including demographic information and medical conditions, such as HAM-related symptoms and medications. This study analyzed data at enrollment in the HAM-net. Of the 558 patients enrolled in the HAM-net between April 1, 2012 and December 31, 2018, 538 patients who had SF-36 data were included in this study (Figure 1). The Bioethics Committee of St. Marianna University School of Medicine approved this study (Approval ID no. 2044). All participants provided written informed consent.

### Evaluation of Health-Related Quality of Life

The patients included in this study answered the Japanese standardized version of the SF-36, version 2 (20, 21). The SF-36, which includes 36 questions, comprises the following eight subscales: physical functioning (PF), limitations on role functioning because of physical health (role physical, RP), bodily pain (BP), general health perception (GH), vitality (VT), social functioning (SF), limitations on role functioning because of emotional problems (role emotional, RE), and mental health (MH). We evaluated the subscales of the SF-36 using the norm-based scoring method based on the national standard value. The norm-based score is standardized with a mean value of 50 and standard deviation (SD) of 10, with higher values indicating better HRQoL. Scores of less than 50 indicate that the HRQoL is worse than that of the general Japanese population. Moreover, we calculated the SF-6D score from the SF-36. The SF-6D is a preference-based HRQoL score for economic evaluation, ranging from 0 to 1, with higher values indicating better HRQoL (9).

### Valuables Extracted From the HAM-net

All data analyzed in this study were collected via telephone interviews conducted at the time of enrollment in the HAM-net. In addition to the SF-36 scores, we extracted data on age and sex as well as medical data regarding the major symptoms of HAM at enrollment, including gait dysfunction, sensory dysfunction in the legs (pain and numbness), urinary dysfunction, and constipation. Gait dysfunction was evaluated using the Osame Motor Disability Score (OMDS), a scale specific to HAM (Table 1) (19). The OMDS ranges from 0 to 13, with higher scores indicating greater walking disability. We divided the OMDS into four levels (i.e., 0–4, 5, 6, and 7–13) to assess the relationship between the OMDS and HRQoL.

• OMDS 0–4: patients who can walk without walking aid.
• OMDS 5: patients who can walk more than 10 m with unilateral support.
• OMDS 6: patients who can walk more than 10 m with bilateral support.
• OMDS 7–13: patients who cannot walk 10 m with bilateral support, including those who cannot walk at all.

**TABLE 1:** Osame motor disability score.

Grade	Motor disability
0	No walking or running abnormalities
1	Normal gait but runs slowly
2	Abnormal gait (stumbling, stiffness)
3	Unable to run
4	Needs handrail to climb stairs
5	Needs a cane (unilateral support) to walk
6	Needs bilateral support to walk
7	Can walk 5–10 m with bilateral support
8	Can walk 1–5 m with bilateral support
9	Cannot walk, but able to crawl
10	Cannot crawl, but able to move using arms
11	Cannot move around, but able to turn over in bed
12	Cannot turn over in bed
13	Cannot even move toes

Leg pain and numbness were reported by patients as absent, occasional, or persistent. Urinary dysfunction was evaluated using the HAM-bladder dysfunction severity grade (HAM-BDSG) (22). The HAM-BDSG comprises four grades (grades 0–III) according to the dependency on urinary catheters and the presence or absence of lower urinary tract symptoms or medications.

• Grade 0 (HAM-BDSG 0): patients who do not use urinary catheters, have no lower urinary tract symptoms, and take no medications for lower urinary tract symptoms.
• Grade I (HAM-BDSG I): patients who do not use urinary catheters but have lower urinary tract symptoms or take medications.
• Grade II (HAM-BDSG II): patients who require intermittent catheters.
• Grade III (HAM-BDSG III): patients who use indwelling catheters.

The presence or absence of constipation was reported by patients without defining constipation. Data on the use of oral laxatives, manual extraction, enemas, and corticosteroids were also extracted. Of the extracted data, only the HAM-BDSG data of eight patients (1.5%) were missing; they were excluded from the HAM-BDSG analyses.

### Outcomes

The outcomes of interest were as follows: (1) HRQoL evaluated using the SF-6D and SF-36 subscales in patients with HAM and (2) impact of HAM-related symptoms on HRQoL.

### Statistical Analysis

Patient characteristics were reported as means and SDs or medians with interquartile ranges (IQRs) for continuous variables and frequencies for categorical variables.

We used the unpaired *t*-test to compare the SF-6D scores of patients with HAM against the national population norm reported by Shiroiwa et al. (23). This report includes SF-6D scores classified according to the age category; however, it did not include an overall population score. Therefore, we compared the SF-6D score of our whole cohort with that of the 60–69 years age category in the general population [mean (SD) score: 0.674 (0.128), *n* = 199], because the mean age of our patients was 62.0 years. The one-sample *t*-test was used to compare the SF-36 subscale scores with the national standard of 50.

We estimated the associations of the SF-6D and SF-36 subscale scores with HAM-related symptoms using a general linear model. We included the following predetermined covariates for multivariate adjustment: age (age categories: 39 years or younger, 40–49 years, 50–59 years, 60–69 years, and 70 years or older), sex, OMDS (0–4, 5, 6, and 7–13), leg pain (absent, occasional, and persistent), leg numbness (absent, occasional, and persistent), HAM-BDSG (Grade 0, I, II, and III), and constipation [absent, present (controlled with or without oral laxatives), and present (needs manual extraction or enemas)]. All variables were treated as categorical. No evidence for multicollinearity problems between covariates were found using the variance inflation factor test (less than 5).

All statistical analyses were performed using Statistical Package for the Social Sciences (version 25; IBM Corporation, Armonk, NY, United States). Two-sided *p*-values of less than 0.05 were used to denote statistical significance.

## Results

### Patient Characteristics

Table 2 summarizes the patient characteristics according to the OMDS category (*n* = 538). The mean age and disease duration were 62.0 and 16.5 years, respectively. Among the patients under study, 74.7% were females. The median grade of OMDS was 5 (IQR, 4–6) with a wide distribution from OMDS 0–4 to OMDS 7–13. The prevalence of leg pain was 42.7% (occasional, 20.6%; persistent, 22.1%), and that of leg numbness was 67.1% (occasional, 19.7%; persistent, 47.4%). Regarding bladder dysfunction, 65.5% of the patients had lower urinary tract symptoms and/or medications (HAM-BDSG I). Intermittent (HAM-BDSG II) and indwelling catheters (HAM-BDSG III) were used in 23.6 and 3.0%, respectively. Among the study population, 77.9% had constipation and 9.3% required manual extraction or enemas. The frequency and severity of HAM-related symptoms increased with worsening OMDS.

**TABLE 2:** Patient characteristics.

Characteristic	All (*n* = 538)	OMDS 0–4 (*n* = 144)	OMDS 5 (*n* = 178)	OMDS 6 (*n* = 96)	OMDS 7–13 (*n* = 120)
Age at interview (years), mean (SD)	62.0 (10.8)	58.3 (11.4)	61.4 (10.7)	65.8 (9.4)	64.4 (9.8)
Age at disease onset (years), mean (SD)	45.4 (14.8)	47.6 (14.1)	46 (14.6)	46.2 (14.6)	41.3 (15.3)
Disease duration (years), mean (SD)	16.5 (11.6)	10.6 (8.8)	15.4 (10.8)	19.6 (11.2)	23 (12.0)
Female sex, n (%)	402 (74.7)	96 (66.7)	139 (78.1)	71 (74.0)	96 (80.0)
OMDS, median (IQR)	5 (4–6)	4 (3–4)	5	6	9 (8–10)
OMDS 0–4, n (%)	144 (26.8)	–	–	–	–
OMDS 5, n (%)	178 (33.1)	–	–	–	–
OMDS 6, n (%)	96 (17.8)	–	–	–	–
OMDS 7–13, n (%)	120 (22.3)	–	–	–	–
**Leg pain**					
Absent, n (%)	308 (57.2)	92 (63.9)	107 (60.1)	52 (54.2)	57 (47.5)
Occasional, n (%)	111 (20.6)	31 (21.5)	30 (16.9)	22 (22.9)	28 (23.3)
Persistent, n (%)	119 (22.1)	21 (14.6)	41 (23.0)	22 (22.9)	35 (29.2)
**Leg numbness**					
Absent, n (%)	177 (32.9)	46 (31.9)	72 (40.4)	30 (31.3)	29 (24.2)
Occasional, n (%)	106 (19.7)	39 (27.1)	30 (16.9)	12 (12.5)	25 (20.8)
Persistent, n (%)	255 (47.4)	59 (41.0)	76 (42.7)	54 (56.3)	66 (55.0)
**HAM-BDSG**					
Grade 0, n (%)	42 (7.9)	23 (16.0)	16 (9.0)	2 (2.1)	1 (0.9)
Grade I, n (%)	347 (65.5)	103 (71.5)	121 (68.4)	69 (71.9)	54 (47.8)
Grade II, n (%)	125 (23.6)	18 (12.5)	38 (21.5)	22 (22.9)	47 (41.6)
Grade III, n (%)	16 (3.0)	0 (0)	2 (1.1)	3 (3.1)	11 (9.7)
Constipation, n (%)	419 (77.9)	101 (70.1)	135 (75.8)	75 (78.1)	108 (90.0)
Oral laxatives, n (%)	355 (66.0)	90 (62.5)	117 (65.7)	65 (67.7)	83 (69.2)
Manual extraction or enemas, n (%)	50 (9.3)	4 (2.8)	12 (6.7)	9 (9.4)	25 (20.8)
Corticosteroid treatment, n (%)	231 (42.9)	55 (38.2)	87 (48.9)	46 (47.9)	43 (35.8)

*HAM-BDSG, HAM-bladder dysfunction severity grade; OMDS, Osame Motor Disability Score.*

### The Short Form 6-Dimension and Short Form-36 Subscale Scores in Patients With HTLV-1-Associated Myelopathy

**FIGURE 2:**
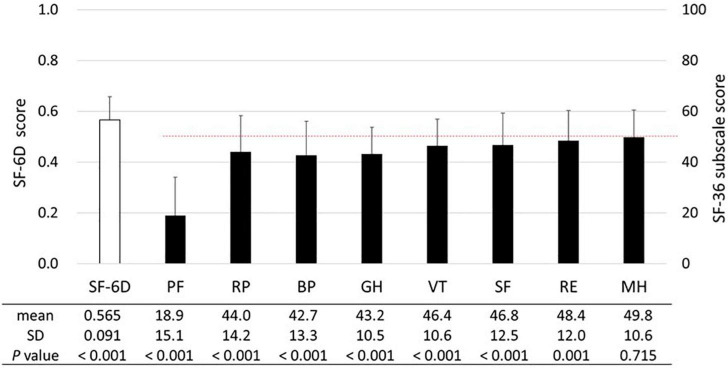
SF-6D and SF-36 subscales. The means and standard deviations (SDs) of the SF-6D and SF-36 subscale scores are presented (*n* = 538). The dashed line (50 points) represents the mean SF-36 subscale score among the general Japanese population. The unpaired *t*-test was used to compare the SF-6D score of patients with HAM to the national population norm reported by Shiroiwa et al. [mean (SD) score in the 60–69 years age: 0.674 (0.128)] (23). The SF-36 subscale scores were compared with the national standard of 50 using the one-sample *t*-test. Abbreviations of SF-36 subscales: BP, bodily pain; GH, general health perception; MH, mental health; PF, physical functioning; RE, role emotional; RP, role physical; SF, social functioning; VT, vitality.

The SF-6D and SF-36 subscale scores are shown in Figure 2. The mean SF-6D score of 0.565 was significantly lower (*p* < 0.001) than the Japanese population norm (mean score in the 60–69 years age group: 0.674) (23). The SF-36 subscale scores were significantly lower than the national standard of 50 (*p* ≤ 0.001), except for the MH score (mean score, 49.8; *p* = 0.715). While subscales associated with physical health (i.e., PF, RP, BR, and GH) tended to be lower than those associated with mental health (i.e., VT, SF, RE, and MH), the PF score was the lowest (mean score, 18.9).

### Impact of HTLV-1-Associated Myelopathy-Related Symptoms on the Short Form 6-Dimension

**FIGURE 3:**
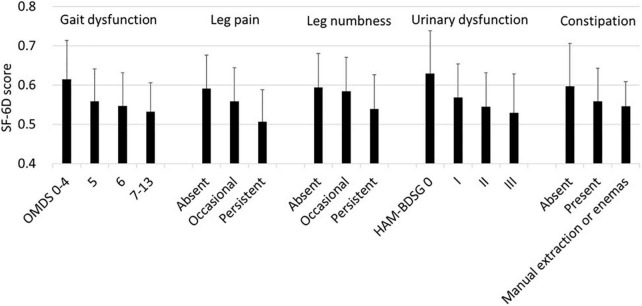
SF-6D according to HAM-related symptoms. The SF-6D score is expressed as mean and standard deviation (*n* = 538). HAM-BDSG, HAM-bladder dysfunction severity grade; OMDS, Osame Motor Disability Score.

First, we analyzed the association of the SF-6D score with each HAM-related symptom. In all symptoms, the SF-6D score decreased with worsening (Figure 3). Because patients had multiple symptoms, we estimated the associations using a multivariate model while adjusting for age and sex (Table 3). All symptoms were significantly associated with a decrease in the SF-6D score. The influence of the OMDS (coefficients vs. OMDS 4: OMDS 5, −0.050; OMDS 6, −0.057; and OMDS 7–13, −0.060; *p* < 0.001 for all) and persistent leg pain (coefficient of persistent pain vs. absent: −0.061; *p* < 0.001) was greater than that of the other symptoms.

**TABLE 3:** Association between the SF-6D and HAM-related symptoms.

Variable	β	95% CI	*P* value
Intercept	**0.673**	**0.642, 0.705**	**<0.001**
**Age (years)**			
20–39	−0.003	−0.043, 0.037	0.889
40–49	0.008	−0.018, 0.034	0.535
50–59 (reference)	Ref		
60–69	0.015	−0.002, 0.032	0.089
70+	0.015	−0.004, 0.034	0.125
**Sex**			
Male (vs. female)	−0.002	−0.018, 0.014	0.826
**OMDS (reference: OMDS 0–4)**			
5	−**0.050**	−**0.068,**−**0.032**	**<0.001**
6	−**0.057**	−**0.078,**−**0.035**	**<0.001**
7–13	−**0.060**	−**0.081,**−**0.038**	**<0.001**
**Leg pain (reference: absent)**			
Occasional	−**0.029**	−**0.046,**−**0.011**	**0.002**
Persistent	−**0.061**	−**0.079,**−**0.042**	**<0.001**
**Leg numbness (reference: absent)**			
Occasional	−0.012	−0.031, 0.008	0.241
Persistent	−**0.029**	−**0.046,**−**0.012**	**0.001**
**HAM-BDSG (reference: Grade 0)**			
Grade I	−**0.028**	−**0.054,**−**0.001**	**0.039**
Grade II	−**0.037**	−**0.067,**−**0.007**	**0.015**
Grade III	−0.036	−0.085, 0.013	0.150
**Constipation (reference: absent)**			
Present, controlled with or without oral laxatives	−**0.018**	−**0.035,**−**0.001**	**0.042**
Present, needs manual extraction or enemas	−0.010	−0.039, 0.019	0.513

β *coefficients (β) and 95% confidential intervals (95% CIs) were calculated using a general linear model to estimate the association between the SF-6D and HAM-related symptoms. Statistically significant coefficients (p < 0.05) are shown in bold. HAM-BDSG, HAM-bladder dysfunction severity grade; OMDS, Osame Motor Disability Score.*

### Impact of HTLV-1-Associated Myelopathy-Related Symptoms on the Short Form-36 Subscales

**FIGURE 4:**
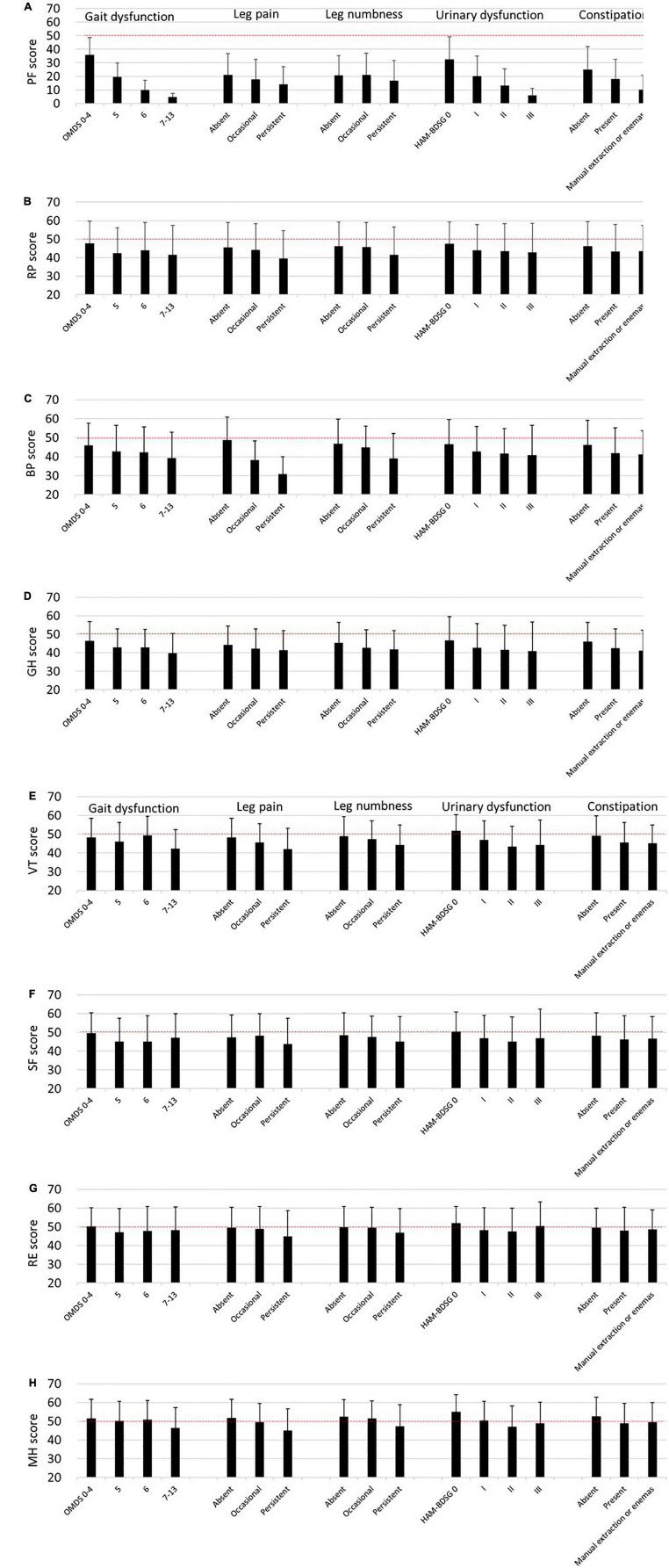
SF-36 subscales according to HAM-related symptoms. The SF-36 subscale scores are expressed as means and standard deviations (*n* = 538). The dashed lines (50 points) indicate the mean score of the SF-36 subscales among the general Japanese population. **(A)** Physical functioning (PF), **(B)** role physical (RP), **(C)** bodily pain (BP), **(D)** general health perception (GH), **(E)** vitality (VT), **(F)** social functioning (SF), **(G)** role emotional (RE), **(H)** mental health (MH). HAM-BDSG, HAM-bladder dysfunction severity grade; OMDS, Osame Motor Disability Score.

Similar to the SF-6D, the SF-36 subscale scores decreased as the symptoms worsened (Figure 4). However, the tendency was weaker in the mental health components than in the physical health components.

Next, we estimated the influence of the symptoms on the SF-36 subscales using multivariate analysis (Table 4). Compared with OMDS 0–4, OMDS 5 or higher was significantly associated with lower scores in PF, RP, GH, and SF. BP and VT scores significantly decreased only in OMDS 7–13. In contrast, RE and MH were not associated with OMDS. Occasional leg pain was significantly associated with lower BP and VT scores. Moreover, persistent pain was associated with a decrease in PF, RP, BP, VT, RE, and MH scores. While occasional numbness was associated only with lower GH scores, persistent numbness was associated with a decrease in RP, GH, VT, SF, and MH scores. Collectively, sensory disturbance in the legs (pain and numbness) was associated with all subscales. HAM-BDSG I was associated with lower PF and VT scores. Furthermore, HAM-BDSG II was associated with a decrease in PF, GH, VT, and MH scores. Constipation was associated only with lower PF scores.

**TABLE 4:** Association between SF-36 subscales and HAM-related symptoms.

	PF			RP			BP			GH		
Variable	β	95% CI	*P* value	β	95% CI	*P* value	β	95% CI	*P* value	β	95% CI	*P* value
Intercept	**41.8**	**38, 45.6**	**<0.001**	**51.2**	**45.7, 56.7**	**<0.001**	**51.0**	**46.6, 55.3**	**<0.001**	**53.6**	**49.6, 57.6**	**<0.001**
**Age (years)**												
20–39	1.2	−3.6, 6	0.623	−2.6	−9.7, 4.4	0.467	2.2	−3.4, 7.8	0.445	−3.1	−8.2, 2	0.229
40–49	1.2	−1.9, 4.2	0.465	−1.5	−6, 3.1	0.528	1.6	−2, 5.2	0.391	−1.7	−5, 1.6	0.309
50–59 (reference)	Ref			Ref			Ref			Ref		
60–69	1.4	−0.7, 3.4	0.200	1.0	−2, 4	0.512	0.0	−2.4, 2.5	0.974	0.0	−2.2, 2.2	0.993
70+	0.0	−2.3, 2.3	0.996	3.0	−0.3, 6.4	0.078	0.0	−2.6, 2.7	0.979	0.6	−1.8, 3.1	0.613
**Sex**												
Male (vs. female)	−0.4	−2.3, 1.5	0.668	1.5	−1.2, 4.3	0.272	**2.9**	**0.7, 5.1**	**0.009**	−1.4	−3.4, 0.6	0.177
**OMDS (reference: OMDS 0–4)**												
5	−**15.8**	−**18,**−**13.7**	**<0.001**	−**4.9**	−**8,**−**1.8**	**0.002**	−1.9	−4.4, 0.5	0.123	−**3.7**	−**5.9,**−**1.4**	**0.002**
6	−**25.0**	−**27.5,**−**22.4**	**<0.001**	−3.7	−7.5, 0	0.050	−1.4	−4.4, 1.6	0.357	−**3.4**	−**6.1,**−**0.7**	**0.013**
7–13	−**28.9**	−**31.4,**−**26.4**	**<0.001**	−**5.5**	−**9.2,**−**1.8**	**0.004**	−**3.5**	−**6.4,**−**0.5**	**0.020**	−**4.9**	−**7.6,**−**2.2**	**<0.001**
**Leg pain (reference: absent)**												
Occasional	−1.9	−4, 0.2	0.076	−1.0	−4.1, 2.1	0.541	−**9.9**	−**12.3,**−**7.4**	**<0.001**	−1.5	−3.8, 0.7	0.187
Persistent	−**2.9**	−**5.2,**−**0.7**	**0.010**	−**3.6**	−**6.9,**−**0.3**	**0.032**	−**16.1**	−**18.7,**−**13.5**	**<0.001**	−1.2	−3.6, 1.2	0.335
**Leg numbness (reference: absent)**												
Occasional	−0.9	−3.3, 1.4	0.425	−0.5	−3.9, 2.9	0.767	−0.9	−3.7, 1.8	0.497	−**2.7**	−**5.2,**−**0.2**	**0.032**
Persistent	−0.4	−2.4, 1.6	0.669	−**3.5**	−**6.4,**−**0.5**	**0.021**	−2.0	−4.3, 0.4	0.103	−**2.6**	−**4.7,**−**0.5**	**0.017**
**HAM-BDSG (reference: Grade 0)**												
Grade I	−**3.7**	−**6.9,**−**0.6**	**0.021**	−1.5	−6.1, 3.1	0.525	0.5	−3.2, 4.2	0.801	−2.6	−6, 0.8	0.132
Grade II	−**5.1**	−**8.7,**−**1.6**	**0.005**	−0.5	−5.7, 4.8	0.859	0.9	−3.3, 5.1	0.669	−**6.2**	−**10.1,**−**2.4**	**0.001**
Grade III	−5.2	−11, 0.6	0.080	−0.2	−8.8, 8.3	0.959	2.9	−3.9, 9.7	0.399	−0.3	−6.5, 5.9	0.919
**Constipation (reference: absent)**												
Present, controlled with or without oral laxatives	−**2.5**	−**4.5,**−**0.5**	**0.017**	−1.6	−4.6, 1.4	0.305	−2.0	−4.4, 0.4	0.102	−1.9	−4.1, 0.3	0.084
Present, needs manual extraction or enemas	−2.9	−6.4, 0.5	0.097	−0.4	−5.4, 4.7	0.889	−1.9	−5.9, 2.1	0.346	−1.1	−4.7, 2.6	0.573

	**VT**			**SF**			**RE**			**MH**		
				
**Variable**	**β**	**95% CI**	***P* value**	**β**	**95% CI**	***P* value**	**β**	**95% CI**	***P* value**	**β**	**95% CI**	***P* value**

Intercept	**56.3**	**52.3, 60.3**	**<0.001**	**53.5**	**48.6, 58.4**	**<0.001**	**53.1**	**48.4, 57.8**	**<0.001**	**58.9**	**54.9, 62.9**	**<0.001**
**Age (years)**												
20–39	−2.1	−7.2, 3	0.417	−1.8	−8, 4.4	0.574	1.2	−4.8, 7.2	0.700	−1.7	−6.8, 3.4	0.514
40–49	−1.8	−5.1, 1.5	0.276	−0.4	−4.4, 3.7	0.863	2.2	−1.6, 6.1	0.258	−0.7	−4, 2.6	0.664
50–59 (reference)	Ref			Ref			Ref			Ref		
60–69	−0.9	−3.1, 1.3	0.420	1.9	−0.8, 4.5	0.169	0.1	−2.5, 2.7	0.930	−1.6	−3.8, 0.6	0.161
70+	−0.9	−3.3, 1.5	0.461	**4.8**	**1.8, 7.8**	**0.002**	1.4	−1.5, 4.2	0.346	−1.0	−3.5, 1.4	0.420
**Sex**												
Male (vs. female)	−0.6	−2.6, 1.4	0.536	−1.7	−4.2, 0.7	0.167	0.7	−1.7, 3	0.573	−0.5	−2.6, 1.5	0.596
**OMDS (reference: OMDS 0–4)**												
5	−1.8	−4.1, 0.4	0.115	−**4.3**	−**7.1,**−**1.6**	**0.002**	−2.3	−5, 0.3	0.087	−0.7	−3, 1.6	0.542
6	2.2	−0.5, 4.9	0.107	−**4.9**	−**8.2,**−**1.6**	**0.004**	−1.7	−4.9, 1.4	0.283	0.7	−2.1, 3.4	0.635
7–13	−**4.0**	−**6.6,**−**1.3**	**0.004**	−1.4	−4.7, 1.9	0.404	−0.5	−3.7, 2.6	0.749	−2.7	−5.4, 0	0.050
**Leg pain (reference: absent)**												
Occasional	−**2.3**	−**4.5,**−**0.1**	**0.043**	1.2	−1.6, 3.9	0.398	−0.7	−3.3, 2	0.618	−1.9	−4.1, 0.4	0.104
Persistent	−**4.4**	−**6.8,**−**2**	**<0.001**	−2.1	−5, 0.7	0.146	−**3.8**	−**6.6,**−**1**	**0.007**	−**5.0**	−**7.4,**−**2.6**	**<0.001**
**Leg numbness (reference: absent)**												
Occasional	−1.2	−3.7, 1.3	0.348	−1.8	−4.8, 1.2	0.243	−0.8	−3.7, 2.1	0.596	−0.8	−3.3, 1.7	0.553
Persistent	−**2.7**	−**4.8,**−**0.5**	**0.014**	−**2.9**	−**5.5,**−**0.3**	**0.030**	−1.9	−4.4, 0.7	0.147	−**2.8**	−**4.9,**−**0.6**	**0.011**
**HAM-BDSG (reference: Grade 0)**												
Grade I	−**3.4**	−**6.8,**−**0.1**	**0.045**	−2.4	−6.5, 1.7	0.255	−2.1	−6.1, 1.8	0.288	−2.5	−5.9, 0.8	0.141
Grade II	−**5.7**	−**9.5,**−**1.9**	**0.003**	−4.0	−8.6, 0.7	0.093	−2.7	−7.1, 1.8	0.242	−**4.7**	−**8.5,**−**0.9**	**0.016**
Grade III	−3.3	−9.5, 2.9	0.292	−3.1	−10.6, 4.4	0.420	0.5	−6.8, 7.7	0.902	−1.8	−8, 4.4	0.575
**Constipation (reference: absent)**												
Present, controlled with or without oral laxatives	−1.7	−3.8, 0.5	0.135	−1.3	−3.9, 1.4	0.353	−0.4	−3, 2.1	0.756	−2.0	−4.2, 0.2	0.071
Present, needs manual extraction or enemas	−0.9	−4.6, 2.7	0.617	−0.4	−4.9, 4.1	0.861	0.4	−3.9, 4.7	0.860	−0.3	−4, 3.4	0.860

β *coefficients (β) and 95% confidential intervals (95% CIs) were calculated using a general linear model to estimate the association between SF-36 subscales and HAM-related symptoms. Bold values represent p-values of less than 0.05. Abbreviations of the SF-36 subscales: BP, bodily pain; GH, general health perception; MH, mental health; PF, physical functioning; RE, role emotional; RP, role physical; SF, social functioning; VT, vitality. HAM-BDSG, HAM-bladder dysfunction severity grade; OMDS, Osame Motor Disability Score.*

## Discussion

In this study, we evaluated the HRQoL of Japanese patients with HAM for an average disease duration of 16.5 years. Most patients had not only gait dysfunction but also sensory disturbance, urinary dysfunction, and constipation. In this study, the SF-6D and SF-36 subscale scores were lower than the population standards. Multivariate analyses demonstrated that all these symptoms were significantly associated with worse HRQoL.

To our knowledge, no studies have reported on the SF-6D score in patients with HAM; however, the poor scores found in this study were consistent with those reported in a study conducted in Brazil and the United Kingdom, which used the EQ-5D index as a health-related utility value (18). We considered that the decrease in the SF-6D score was clinically meaningful because it was as large as a minimal important difference of 0.05–0.1 (23). Moreover, all symptoms analyzed were significantly associated with lower SF-6D scores in the multivariate analysis. The coefficients in OMDS 5 or higher (vs. OMDS 0–4) and persistent leg pain (vs. absent) were remarkably large and met the minimal important difference, indicating that the management of motor and sensory dysfunctions in the legs has a strong impact on HRQoL. However, caution is required in interpreting the results of this study. Although the SF-36 comprises questions directly reflecting gait function (PF) and pain (BP), no questions focus on other HAM-related symptoms. Hence, compared to gait function and pain, the SF-36 indirectly evaluates the effects of leg numbness, urinary dysfunction, and constipation on HRQoL, which may lead to an underestimation of their burden. Therefore, recognizing that all major symptoms of HAM can significantly affect HRQoL is important.

In this study, the SF-36 subscale scores other than the MH score were below the national average, and the subscales related to physical health were lower than those related to mental health. PF was worst of all the subscales. These findings are consistent with those reported in a Brazilian study (15), suggesting that poor physical health is a characteristic feature of HAM. Regarding the association between OMDS and the subscale scores, PF is naturally and strongly correlated with OMDS because PF mainly reflects mobility. Moreover, OMDS 5 or higher was significantly associated with a decrease in RP, GH, and SF scores; however, the differences in the coefficients between OMDS 5, 6, and 7–13 were relatively small, indicating that there is a large gap in HRQoL between OMDS 0–4 and 5. As a result, maintaining gait function better than OMDS 5 may be important for better HRQoL and can be a therapeutic goal for patients with HAM. In general, gait dysfunction caused by HAM progresses slowly, requiring 5–10 years on average to reach OMDS 5 (19, 24). However, some patients with high inflammatory activity in the spinal cord experience faster deterioration, with OMDS 5 or higher within 2 years after the disease onset (25). Thus, early diagnosis is important for preventing loss of treatment opportunities. Although there is no curative treatment for HAM, evidence suggests that corticosteroid therapy is effective in maintaining gait function (26). The international guideline for HAM recommends the use of corticosteroids based on the rate of disease progression (5). Early diagnosis, evaluation of progression, and treatment initiation at the appropriate time are all essential in the treatment of HAM.

In addition, we found that sensory disturbance and urinary dysfunction affect HRQoL in patients with HAM. First, persistent leg pain and numbness were associated with lower RP scores, indicating that not only gait dysfunction but also sensory disturbance hamper daily activity in patients with HAM. Second, although the OMDS had no significant influence on MH, persistent leg pain and numbness as well as the use of intermittent catheters (HAM-BDSG II) were significantly associated with lower MH scores. These findings are consistent with those reported in recent HRQoL studies in patients with multiple sclerosis and neuromyelitis optica, both of which are neuroinflammatory diseases causing symptoms similar to those in HAM. They reported that not gait dysfunction but chronic pain, including numbness, was associated with depression and mental health satisfaction (27, 28). Third, leg pain, numbness, and urinary dysfunction were also associated with lower GH and VT scores. Intermittent catheterization had the greatest impact on GH and VT of all the symptoms analyzed. In an HRQoL study involving patients who developed neurogenic bladder dysfunction after radical hysterectomy, GH, VT, and MH scores were lower in patients on intermittent catheterization than those in patients with spontaneous voiding, despite similar clinical backgrounds other than the use of intermittent catheters (29). The negative effects of urinary dysfunction on GH, VT, and MH observed in the present study are supported by this report. Collectively, sensory and urinary dysfunctions in patients with HAM should be carefully managed to maintain better HRQoL both physically and mentally.

In this study, the use of indwelling catheters or manual extraction or enemas was not associated with the SF-36 subscale scores despite being the most severe form of bladder and bowel dysfunction. Although the reason is unclear, “response shift” may be one of the reasons. Response shift is a psychological process occurring in individuals with deteriorating health, in which internal standards and values change to adapt to a new situation (30, 31). Longitudinal studies are required to understand the changes in HRQoL over time in patients with HAM.

This study has several limitations. First, HRQoL is influenced by several factors not included in our analysis, such as education, employment status, and comorbidities (23). Moreover, we did not evaluate the intensity of the symptoms; HAM causes many other symptoms, including low back pain and spasticity (4). Therefore, we could not completely adjust symptom severity and potential confounders. Second, the coefficients only indicate associations, and therefore, may not necessarily mean the impact of the symptoms. Third, all symptoms were assessed using information collected through telephone interviews. Although telephone interviews were conducted uniformly by trained nurses and coordinators to collect reliable information, the accuracy of these assessments may be lower than that of a physical examination. In addition, because leg pain, numbness, lower urinary tract symptoms, and constipation are common even in the general population, we could not conclude that these symptoms were caused by HAM. Finally, because we only included Japanese patients, generalizability to other populations may be limited.

## Conclusion

We demonstrated that HRQoL of patients with HAM was poorer than that in the general population and was associated with various symptoms, including motor, sensory, and bladder and bowel dysfunctions. Therefore, these patients should be comprehensively monitored and managed to achieve better HRQoL.

## Data Availability Statement

The original contributions presented in the study are included in the article/supplementary material, further inquiries can be directed to the corresponding author.

## Ethics Statement

The studies involving human participants were reviewed and approved by the Bioethics Committee of St. Marianna University School of Medicine. The patients/participants provided their written informed consent to participate in this study.

## Author Contributions

MK, JY, TS, NY, NA, SA, KT, AT, and YY contributed to the conception and design of the study. MK, JY, and KT conducted the statistical analysis. MK, JY, TS, NY, NA, SA, KT, EH, TW, AC-R, MN, YA, KK, TK, SS, HM, AK, SI, AT, and YY analyzed and interpreted the results and critically revised the manuscript. MK, JY, and YY drafted the manuscript. All authors read and approved the final version of the manuscript to be submitted.

## Conflict of Interest

SA was employed by the LSI Medience Co., Tokyo, Japan. The remaining authors declare that the research was conducted in the absence of any commercial or financial relationships that could be construed as a potential conflict of interest.

## Publisher’s Note

All claims expressed in this article are solely those of the authors and do not necessarily represent those of their affiliated organizations, or those of the publisher, the editors and the reviewers. Any product that may be evaluated in this article, or claim that may be made by its manufacturer, is not guaranteed or endorsed by the publisher.
